# Mechanical ventilation and clinical practice heterogeneity in intensive care units: a multicenter case-vignette study

**DOI:** 10.1186/2110-5820-4-2

**Published:** 2014-02-01

**Authors:** Yên-Lan Nguyen, Elodie Perrodeau, Bertrand Guidet, Ludovic Trinquart, Jean-Christophe M Richard, Alain Mercat, Philippe Jolliet, Philippe Ravaud, Laurent Brochard

**Affiliations:** 1 AP-HP, Cochin Academic Hospital, Surgical ICU, F-75014 Paris, France; 2 UPMC University Paris 06, UMR_S 707, Sorbonne Universités, F-75013 Paris, France; 3 INSERM, UMR_S 707, F-75011 Paris, France; 4 Paris-Descartes University, UMR_S 738, Sorbonne Universités, F-75014 Paris, France; 5 INSERM, UMR_S 738, French Cochrane Center, F-75001 Paris, France; 6 AP-HP, Saint-Antoine Academic Hospital, Medical ICU, F-75011 Paris, France; 7 ICU, Geneva University Hospital, Geneva, Switzerland; 8 St Michael’s Hospital, Toronto and Interdepartmental Division of Critical care Medicine, University of Toronto, Toronto, Canada; 9 INSERM, UMR_S 955, Team 13, F-94000 Créteil, France; 10 Paris-Est University, UMR_S 955, F-94000 Créteil, France; 11 Medical ICU, Angers Academic Hospital, F-49000 Angers, France; 12 Medical ICU, Lausanne Academic Hospital (CHUV), Lausanne, Switzerland

**Keywords:** Mechanical ventilation, Clinical practice, Volume-outcome, Protocols

## Abstract

**Background:**

Observational studies on mechanical ventilation (MV) show practice variations across ICUs. We sought to determine, with a case-vignette study, the heterogeneity of processes of care in ICUs focusing on mechanical ventilation procedures, and whether organizational patterns or physician characteristics influence practice variations.

**Methods:**

We conducted a cross-sectional multicenter study using the case-vignette methodology. Descriptive analyses were calculated for each organizational pattern and respondent characteristics. An Index of Qualitative Variation (IQV, from 0, no heterogeneity, to a maximum of 1) was calculated.

**Results:**

Forty ICUs from France (N = 33) and Switzerland (N = 7) participated; 396 physicians answered our case-vignettes. There was major heterogeneity of management processes related to MV within and across centers (mean IQV per center 0.51, SD 0.09). We observed the lowest variability (mean IQV per question < 0.4) for questions related to intubation procedure, ventilation of acute respiratory distress syndrome and the use of the semirecumbent position. We observed a high variability (mean IQV per question > 0.6) for questions related to management of endotracheal tube or suctioning, management of sedation and analgesia, and respect of autonomy. Heterogeneity was independent of respondent characteristics and of the presence of written procedures. There was a correlation between the processes associated with the highest variability (mean IQV per question > 0.6) and the annual volume of ICU admission (r = 0.32 (0.01 to 0.58)) and MV (r = 0.38 (0.07 to 0.63)). Within ICUs there was a large heterogeneity regarding knowledge of a local written procedure.

**Conclusions:**

Large clinical practice variations were found among ICUs. High volume centers were more likely to have heterogeneous practices. The presence of a local written procedure or respondent characteristics did not influence practice variation.

## Background

Unwarranted clinical practice variation is common in medicine. Several studies suggest that patients with similar demographic patterns, co-morbidities, diagnoses and severity of illness receive different levels of care depending on when, where or by whom they are treated [1,2]. Some variability may be justified by uncertainty in knowledge, need to individualize patient care and differences in case-mix, and can be related to how compelling individual clinicians find particular information [3]. Unexplained variability in practice could potentially lead to heterogeneous quality and safety in the care of patients.

Mechanical ventilation (MV) is applied to around 30% to 70% of patients admitted in the ICU [4]. Not surprisingly, multicenter observational studies suggest practice variation in MV [5]. Case-mix and ICU organizational patterns such as MV annual volume and processes of care used may account for the variability observed.

Assessment of the heterogeneity in processes of care is not easy to capture accurately. The use of case-vignette is interesting since it is not influenced by case-mix, is easily conducted and not costly. We sought to determine whether heterogeneity exists regarding processes of care associated with MV management through a cross-sectional survey using case vignettes and whether organizational patterns or intensivists’ characteristics influence practice variation. We aimed at assessing the variability existing among the physicians at each center, and also to compare the degree of variability between centers and whether we could find a relationship with some organizational patterns.

## Methods

### Study design and population

We conducted a cross-sectional multicenter survey across ICUs in France and Switzerland, members of the European critical care research REVA network. An invitation to participate to this study was sent to the local coordinators of 48 ICUs belonging to the REVA network. Our study population potentially included all physicians (including those in training) belonging to participating centers. We evaluated physician bedside practices using two case-vignettes (written questionnaire). The main drawbacks of case-vignettes are the absence of control for the conditions in which the respondents answer questions, the Hawthorne effect and the artificial nature of the cases. To minimize some of these effects we asked each center to have their participants respond almost simultaneously during one session that occurred between 5 and 16 September 2011. Some centers held two sessions, when enough respondents could not be present at one single meeting.

### Development of the case-vignette

We used two case-vignettes, written in French. One was that of a 75 year-old man with an acute exacerbation of chronic obstructive pulmonary disease and the other that of a 30 year-old woman suffering from the acute respiratory distress syndrome secondary to H1N1 flu infection. For the two scenarios, participants had to answer 26 closed-ended questions used to assess the processes of care (See Additional file 1).

Questions on intubation involved the preferential method of pre-oxygenation, medications for intubation, intubation site, prevention and management of hypotension following the procedure, and processes used to check endo-tracheal tube position and the measurement of cuff pressure. Questions on ventilator settings involved regular settings for a patient with chronic obstructive pulmonary disease and those for a patient with acute respiratory distress syndrome undergoing invasive MV. Questions concerning sedation-analgesia pertained to timing (start-weaning), pain associated with endo-tracheal suctioning and daily monitoring. Questions on MV liberation and management dealt with the weaning method used, its duration and methods for prevention of laryngeal edema, prevention of desaturation during endotracheal suctioning, medical prescription of restraint, semi-recumbent position, chest X-rays, tracheotomy and plateau pressure monitoring. Questions concerning communication included physician attitude in terms of clinical decision-making (paternalist, autonomist, mixed).

For each question, participants chose among four to six proposed answers, which were based on current national and international recommendations and evidence from published trials. Finally, participants answered questions pertaining to demographical characteristics (age category, gender, professional status), their knowledge of the existence of written procedures (MV liberation, sedation-analgesia recommendations, and lung protective ventilation strategy) or the ICU policy regarding assessment of two incidents associated with MV (ventilator acquired pneumonia, unplanned endo-tracheal self-extubation) in their ICUs. The case-vignettes were first tested among a small group of residents and finally validated by YLN and LB.

### Organizational factors

Each ICU physician director or clinical research coordinator answered a questionnaire describing the ICU organization. The questions concerned the academic status, the number of staffed beds, the nurse-to-patient ratio, the physician-to-patient ratio and the physiotherapist-to-patient ratio. There were questions on the presence of a daily multi-disciplinary round (a round gathering both physicians, nurses and other health care professionals such as respiratory therapist, pharmacist or dietician) and whether there were written procedures or protocols for ventilation for acute respiratory distress syndrome, ventilation liberation, sedation management and prevention of ventilator-associated pneumonia; we also added questions on the existence of local evaluation of the prevalence of self-extubation and ventilator-associated pneumonia.

### Ethics

Our study was a survey of physicians performed on a voluntary basis and did not concern patients or families.

### Statistical analysis

#### Characteristics of the centers and of the respondents

Descriptive analyses were calculated for each organizational pattern (academic status, number of beds, nurse-to-patients ratio, existence of a daily multidisciplinary round, annual number of admissions, annual number of admissions requiring MV) and respondent characteristics (age, gender, function); means (SD) or medians (IQR) were calculated for quantitative variables and frequencies (percentage) for qualitative variables.

#### Variations in processes of care

To assess variation in processes of care, we used the Index of Qualitative Variation (IQV). This coefficient was used to measure the variation among the answers of physicians to each question. The IQV is based on the ratio of the total number of differences in the distribution to the maximum number of possible differences within the same distribution. It was calculated as:

[1 – ∑p_i_^2^] _*_ [K/(K – 1)]

where p_i_ is the proportion of physicians who chose answer *i* among the K proposed answers for a given question [6]. The IQV can take values between 0 and 1, where 0 denotes no variation in practices and 1 means maximum variation in practices, and where 0.5 means the distribution of answers shows 50% of the maximum variation possible.

First, we calculated an IQV for each of the 26 questions in each participating center. Second, we calculated a mean IQV for each of the 26 questions (mean IQV per question) for all the participating centers (see Additional file 1). We will express this value in mean (± SD). According to the distribution of mean IQVs per question (see Additional file 1), we defined questions with lowest or highest variability. A question with an IQV value lower than the first quartile of distribution was considered associated with the lowest variability. Conversely, a question with an IQV value greater than the third quartile of distribution was considered associated with the highest variability. Third, we calculated a mean IQV per center (based on 26 IQVs) to assess the global variability for each center (Additional file 1). We will express this value in mean (± SD). This index allowed us to take into account the correlation among respondents of a same center.

The global variability was compared according to ICU organization patterns. Mean IQV (per center) differences according to teaching status, ICU type, country and presence of a multi-disciplinary round, were tested with a Student *t*-test or an ANOVA. The association between mean IQV per center and total ICU volume, volume of patients mechanically ventilated, was tested using the Pearson coefficient of correlation.

We also tested the association between the practice variation (mean IQV per center) for questions with either an IQV value lower than the lower limit of the first quartile (low heterogeneity) or with an IQV value greater than the upper limit of the third quartile (high heterogeneity), and the annual total ICU volume, the annual volume of mechanically ventilated patients, the annual patients-per-physician and beds-to-physician ratio using the Pearson coefficient of correlation.

Then, for each question, a mean IQV was calculated by modality of the studied respondent characteristics. Distributions of mean IQV per question in each modality were compared by the mean of paired Wilcoxon tests or Friedman tests. This analysis did not take into account the correlation between respondents in a center.

#### Written procedures and reporting of complications

The influence of the presence of a written procedure on variability was tested with the use of Student *t*-tests; for each topic, a mean IQV for questions relative to the studied topic was evaluated. Concordance rates between local coordinators and respondents for the existence of written procedures or for assessment of MV-associated incidents were estimated punctually and per confidence interval by a mixed logistic regression with a random effect on the center to take into account a potential center effect. Analyses were performed using R 2.14 [7].

## Results

### Characteristics of the centers

Forty centers from France (n = 33) and Switzerland (n = 7) participated in the study. Three- quarters of the centers were located in teaching hospitals. Their characteristics are presented in Table 1. The average annual number of patients undergoing MV was 542 ± 355 (58 ± 19% of total admissions).

**Table 1:** Characteristics of the centers

**Centers characteristics**	**N = 40**	
French, N (%)	33	(83)
Swiss, N (%)	7	(18)
Mixed ICUs, N (%)	20	(50)
Medical ICUs, N (%)	15	(40)
Surgical ICUs, N (%)	4	(10)
Multidisciplinary round, N (%)	24	(60)
Number of beds, median (Q1-Q3)	18	(15 to 20)
Patients-to-physician ratio, median (Q1-Q3)	1.4	(1.3 to 2.6)
Patients-to-nurse ratio, median (Q1-Q3)	2.5	(2.5 to 3)
Patients-to-physiotherapist ratio, median (Q1-Q3)	12	(9 to 16)
Total admissions annual volume (annual number of patients), median (Q1-Q3)	849	(609 to 1,070)
Mechanical ventilation annual volume (annual number of patients with MV), median (Q1-Q3)	474	(311 to 614)

Q1-Q3: 25% and 75% percentiles.

### Characteristics of the respondents

Three hundred and ninety-six physicians answered the case-vignettes. Their characteristics are presented in Table 2. The majority (70%) of respondents were under 41 years of age.

**Table 2:** Characteristics of respondents

**Respondents characteristics**	**N = 396**	
French, N (%)	319	(81)
Swiss, N (%)	77	(19)
Male, N (%)	264	(67)
Age category, N (%)		
20 to 30 years old	129	(33)
31 to 40 years old	150	(38)
41 to 50 years old	64	(17)
> 50 years old	48	(12)
Professional status, N (%)		
Senior attending with academic status	51	(13)
Senior attending without academic status	126	(32)
Junior attending	88	(22)
Resident	131	(33)

### Variations in processes of care

An important heterogeneity of processes of care related to MV was found across centers (mean IQV by center 0.50; SD 0.09).

**Figure 1:**
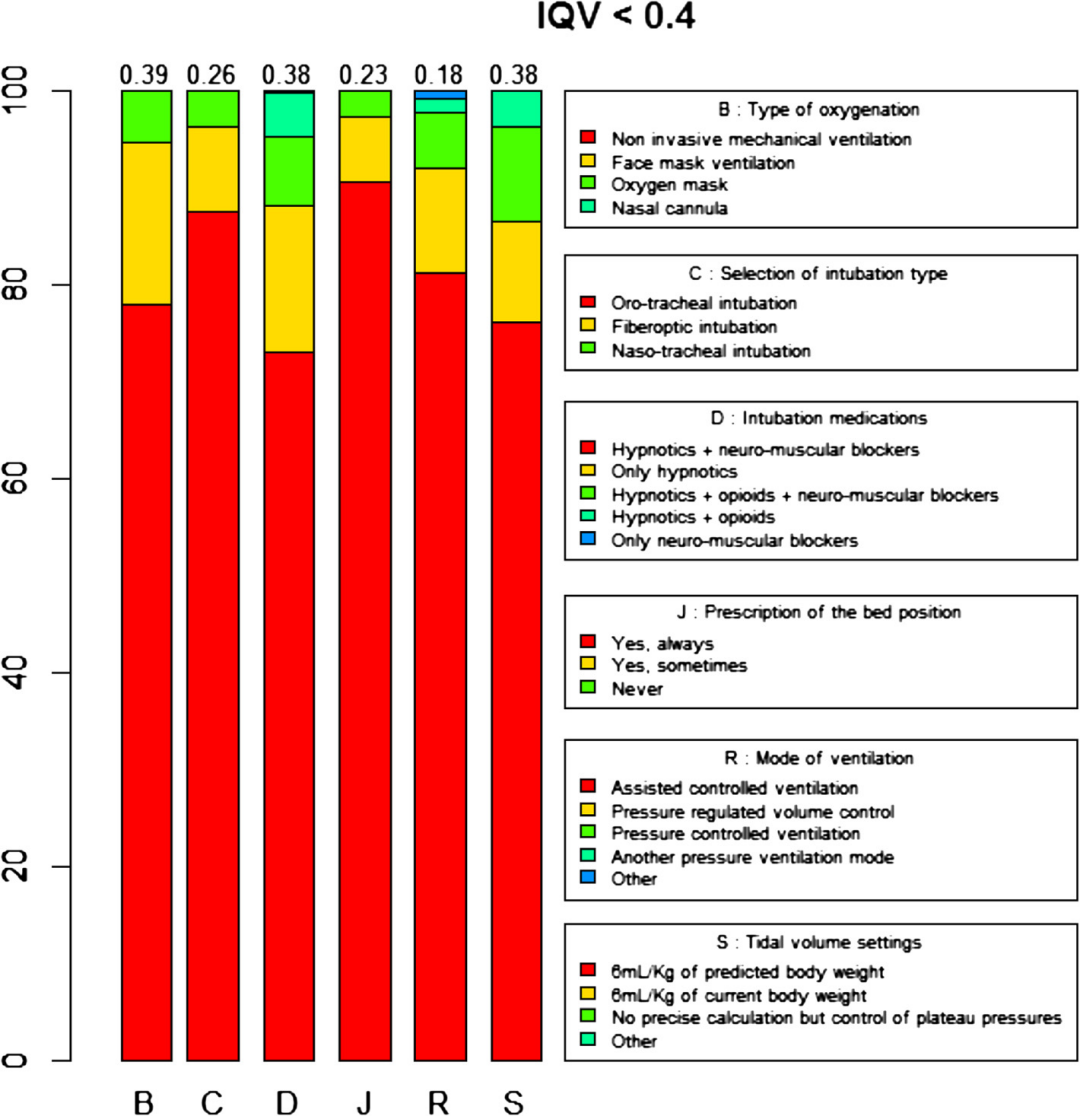
Processes of care associated with lowest practice variation.

We observed the lowest variability (mean IQV per question < 0.4, corresponding to the lower limit of the second quartile) for questions related to the type of pre-oxygenation (mean IQV = 0.39 ± 0.31), the selection of intubation type (mean IQV = 0.26 ± 0.31), intubation medications (mean IQV = 0.38 ± 0.29), the prescription of semirecumbent position (mean IQV = 0.23 ± 0.31), the mode of ventilation (mean IQV = 0.18 ± 0.23) and the tidal volume settings (mean IQV = 0.38 ± 0.32) for patients with acute respiratory distress syndrome (Figure 1). Respondents were more likely to use non invasive ventilation for pre-oxygenation, to perform oro-tracheal intubation, to use an association of hypnotics and neuro-muscular blockers for intubation, to prescribe on a daily basis a semirecumbent position, to select volume controlled ventilation as the preferential setting for patients with chronic obstructive pulmonary disease or acute lung injury undergoing invasive MV and to calculate the tidal volume using the formula of 6 mL/kg of predicted body weight.

**Figure 2:**
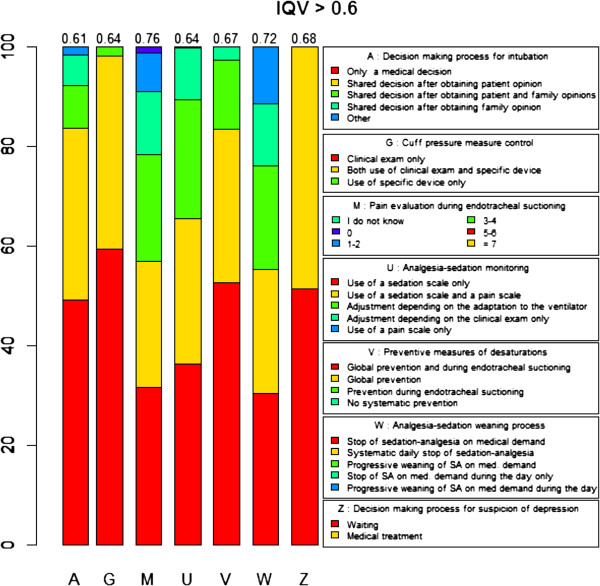
Processes of care associated with highest practice variation.

We observed the highest variability (mean IQV per question > 0.6, corresponding to the upper limit of the third quartile) of processes of care for questions related to the respect of autonomy (mean IQV = 0.61 ± 0.22), control of the endotracheal tube cuff pressure (mean IQV = 0.64 ± 0.20), preventive methods for desaturation and pain evaluation during endo-tracheal suctioning (mean IQV = 0.67 ± 0.18 and 0.76 ± 0.15 respectively), analgesia-sedation monitoring (mean IQV = 0.64 ± 0.23) and sedation weaning (mean IQV = 0.72 ± 0.22) (Figure 2).

### Association between variation in processes of care and organizational factors

There was no correlation between the mean IQVs (per center) and the ICU organization patterns such as teaching status (mean IQV 0.50 ± 0.09 versus 0.51 ± 0.10 for academic non academic, *P* = 0.871), ICU type (mean IQV 0.55 ± 0.10 versus 0.49 ± 0.06 versus 0.51 ± 0.11 for surgical, medical and mixed, *P* = 0.431) or presence of a multidisciplinary round (mean IQV 0.51 ± 0.10 versus 0.50 ± 0.08 for presence or lack of a multidisciplinary round) *P* = 0.648). A higher variability of processes of care was observed in Swiss versus French ICUs (mean IQV 0.57 ± 0.09 versus 0.49 ± 0.09; *P* = 0.048). Total ICU admission volume (r = 0.26 (-0.05; 0.53)) and MV volume (r = 0.19 (-0.14 to 0.48)) were not correlated with practice variation reflected by global IQV. We found no correlation between the questions with lowest variability (mean IQV per question < 0.4) and the total ICU admission volume (r = 0.17 -0.15 to 0.46)) or the MV volume (r = 0.04 (-0.35 to 0.28)). By contrast, the questions with the highest variability (mean IQV per question > 0.6) were associated with the total ICU admission volume (r = 0.32 (0.01 to 0.58)) or the MV volume (r = 0.38 (0.07 to 0.63)) (Table 3).

**Table 3:** Association between questions with lowest practice variation (IQV < 0.4) and organizational factors

**Organizational factor**	**Questions with lower IQV (< 0.4)**		**Questions with higher IQV (> 0.6)**	
	**Correlation coefficient**	**95% CI**	**Correlation coefficient**	**95% CI**
Annual volume of ICU admissions	0.17	(-0.15; 0.46)	0.32	(0.01; 0.58)
Annual volume of MV admissions	0.04	(-0.35; 0.28)	0.38	(0.07; 0.63)
Annual volume of patients per physician	0.19	(-0.13; 0.49)	-0.06	(-0.36; 0.26)
Beds-to-physician ratio	-0.28	(-0.55; 0.03)	-0.10	(-0.40; 0.22)

The presence of a written procedure for ventilation liberation (mean IQV 0.53 ± 0.16 versus 0,45 ± 0.20 for no or existing procedure, *P* = 0.139), sedation management (mean IQV 0.59 ± 0.13 versus 0.63 ± 0.14 for no or existing procedure, *P* = 0.475) or lung protective ventilation strategies (mean IQV 0.40 ± 0.20 versus 0.31 ± 0.16 for no existing procedure, *P* = 0.133) were not correlated with mean IQV on specific questions.

### Association between variation in processes of care and intensivists characteristics

Respondent characteristics such as age (*P* = 0.809), gender (*P* = 0.960), and professional status (*P* = 0.145) were not correlated with IQV per center.

### Written procedures

Less than half of our participating centers had a written procedure for ventilation liberation or for lung protective strategies for acute lung injury (Table 4). There were large variation between the answers of respondents and clinical research coordinators on the existence of written procedures (Table 4).

**Table 4:** Concordance between local coordinators and respondents on the questions on the existence of written procedures and on the measure of incidents associated with mechanical ventilation

	**Prevalence N (%)**		**Rate of good answers % (95% CI)**	
Written procedures				
Ventilation liberation	17	(43)	82	(75 to 87)
Lung protective strategies for acute lung injury	16	(40)	72	(62 to 80)
Sedation-analgesia management	30	(75)	87	(79 to 93)
Prevention of ventilation acquired pneumonia	23	(58)	63	(51 to 73)
Incidents associated with mechanical ventilation				
Ventilator acquired pneumonia	33	(83)	68	(57 to 77)
Unplanned endo-tracheal self-extubation	26	(65)	66	(54 to 76)

### Reports of incidents associated with MV

Thirty-three (83%) centers reported their own rate of ventilator-associated pneumonia and 26 (65%) their rate of unplanned endo-tracheal self-extubation. There were large variation between the answers of respondents and clinical research coordinators on the reports of incidents associated with MV (Table 4).

## Discussion

In this cross-sectional multicenter study, we found a high level of practice variations for processes of care related to MV. This heterogeneity was independent of respondent characteristics and of the presence of written procedures. For questions with high variability, high volume ICUs were more likely to have greater practice variation.

We observed a relative consensus among our respondents regarding the intubation process of a patient with acute respiratory failure and the management of a patient with acute respiratory distress syndrome. This consensus can be considered as based on evidence [8]. The choice of volume controlled ventilation and the use of the 6 mL/kg formula to calculate tidal volume in cases of patients with acute lung injury are concordant with international recommendations and large randomized controlled trials [9-11].

The lack of consensus among physicians for sedation management is interesting since it has been the topic of numerous trials [12-14]. One quarter of the participating units did not use a sedation-management protocol. These results are similar to those of a recent survey conducted in North America [15] and may reflect a gap between research results and practice of critical care medicine [3]. There are several potential reasons. First, this observation might due to a lack of awareness or familiarity with the protocols suggested in the literature. In many ICUs, sedation-management protocols are driven by nurses and physicians may not feel highly involved. Second, this might be due to a lack of self-efficacy of the protocols due to the local ICU organization or a lack of resources. Indeed, the nurse to patient ratio was 0.4 in our sample size, which is low in comparison to the study showing the benefits of the lack of sedation among mechanically ventilated patients (ratio 1:1) [12]. Third, this might be to an attitude of lack of agreement with guidelines in general because they limit autonomy and appear as giving 'cookbook recipes’ [16]. Fourth, the variety of answers observed among physicians about sedation management may just reflect the way people think and make their decisions. Indeed, during the last 30 years, the rational model of judgment has been overtaken by behavioral psychology discoveries suggesting that clinicians, like others, are prone to cognitive biases (such as pattern recognition, indexing keys or gist) leading to systematic and predictable errors [17].

The lack of consensus among physicians for asking patient or family opinion on medical decisions may reflect differences in the vision of the physician and patient relationship among physicians. Indeed, exclusive paternalistic attitude used to be the rule 20 or 30 years ago in France and this contrasts greatly with the autonomist tradition observed in North America. In France, where the majority of respondents work, it is only since 2002 and the promulgation of a new law [18], that the consent of patients has been requested by law before conducting any invasive treatment or procedure. To our knowledge, there is no data on the influence of this law on the way French physicians make their medical decisions. Therefore, it may not be surprising that after ten years, a large practice variation remains.

The presence of a written procedure does not mean that physicians will follow it. We found that the presence of a written procedure on sedation management did not reduce the practice variation. One of the underlying reasons might be the lack of knowledge of the written procedure. Indeed, we found that among ICUs self-reporting a protocol for sedation management, approximately 20% of the physicians were unaware of it. Hence, the presence of written procedures does not mean that there is an ICU culture promoting the benefits of written procedures to reduce practice variation within participating ICUs. Sinuff *et al*. showed in a multicenter qualitative study that the presence of a culture of guidelines within the ICU is key to facilitate clinicians’ adherence to guidelines [19]. Another explanation may be that written procedures apply poorly to complex situations and are rapidly judged useless when addressing complex issues.

The low rate of concordance between the answers of respondents and clinical research coordinators for ventilator-associated pneumonia and unplanned self-extubation prevalence could reflect a lack of quality improvement culture, a communication problem or a lack of confidence in the usefulness of such measures.

Several studies suggest an association between mechanical ventilation volume and patient centered outcomes [20-23]. Kahn *et al*. found that medical critically ill patients undergoing mechanical ventilation had large survival benefits when hospitalized in high volume hospitals (OR mortality = 0.63 (0.50 to 0.79)) [23]. Besides the famous adage 'practice makes perfect’, underlying mechanisms of the volume/outcome relationship remain unclear. This is the first study reporting an association between MV practice variation and ICU MV volume. Interestingly, our data suggest that high volume ICUs are more likely to have heterogeneous practices but only for processes that are not consensual (with the highest IQV variability). One can hypothesize that the larger variety of case-mix present in high volume ICUs may allow high volume intensivists to identify more easily the situations where 'one size does not fit all’ and that may explain the larger practice variation present in high volume ICUs. Then, one way to improve the outcome of patients cared in low volume institutions could be the use of simulation training in order to enhance clinical judgment skills during rare situations.

Our study suffers from several limitations. First, the methods of case-vignettes may not reflect real practices, particularly if physicians answered theoretically rather than based on their actual practice. Second, we could not study the association between practice variation and patient centered outcomes. Third, although we chose two typical situations of critically ill patients undergoing MV, those situations might have been very different from the case-mix of participating centers, thus putting our respondents in uncomfortable positions. Fourth, our results may not reflect French or Swiss practice variation because our study suffers from possible selection bias.

## Conclusions

To conclude, our case-vignette study on processes of care related to MV showed a large practice variation among centers. Heterogeneity of practices was independent of respondent characteristics and of the presence of written procedures. For questions with high variability, higher ICU admissions volume and MV volume were associated with greater practice variation. Further studies are needed to better understand underlying reasons explaining practice variation.

## Abbreviations

ANOVA: analysis of variance; IQV: Index of Qualitative Variation; MV: mechanical ventilation.

## Competing interests

The authors declare that they have no competing interests.

## Authors’ contributions

Drafting of the manuscript: YLN, LB. All authors read and approved the final manuscript.

## Authors’ information

Presented partially by Nguyen YL, Ravaud P, Richard JCM, Mercat A, Guidet B, Brochard L on the behalf of the REVA Network. Mind the gap! The self-reported use of clinical protocols as a quality indicator may be misleading. 2012 ATS International Conference, San Francisco.

## Supplementary Material

Additional file 1 Case-vignettes and mechanical ventilation. Click here for file

## Collaborators

K Asehnoune (Nantes), G Beduneau (Rouen), P Beuret (Roanne), L Brochard (Geneva), L Chalumeau (Villejuif), JD Chiche (Cochin), Y Cohen (Bobigny), F Collet (Saint-Malo), A Combes (Paris), M Darmon (Saint Priest en Jarez), F Decamps (Pau), J Dellamonica (Nice), A Demoule (Paris), J Fichet (Clamart), JP Frat (Poitiers), T Fumeaux (Nyon), S Govind (Fribourg), A Gros (Rennes), C Guérin (Lyon), B Guidet (Paris), C Guitton (Nantes), G Hilbert (Bordeaux), S Jaber (Montpellier), P Jolliet (Lausanne), Q Lu (Paris), A Mercat (Angers), JC Navellou (Besançon), D Osman (Le Kremlin Bicêtre), A Pagnamenta (Mendrisio), L Papazian (Marseille), D Perez (Dole), A Perren (Bellinzona), JP Quenot (Dijon), T Rimmelé (Lyon), L Robriquet (Lille), F Schortgen (Créteil), F Thibaut (Saint Nazaire), A Vieillard Baron (Boulogne), H Zender (Neuchâtel), E Zogheib (Amiens).

## References

[B1] Checkley W Martin GS Brown SM Chang SY Dabbagh O Fremont RD Girard TD Rice TW Howell MD Johnson SB O’Brien J Park PK Pastores SM Patil NT Pietropaoli AP Putman M Rotello L Siner J Sajid S Murphy DJ Sevransky JE United States Critical Illness and Injury Trials Group Critical Illness Outcomes Study Investigators Structure, process, and annual ICU mortality across 69 centers: United States critical illness and injury trials group critical illness outcomes study Crit Care Med 2014 Feb 4 2 344 56 10.1097/CCM.0b013e3182a275d7 24145833 PMC4035482

[B2] Rice TW Morris S Tortella BJ Wheeler AP Christensen MC Deviations from evidence-based clinical management guidelines increase mortality in critically injured trauma patients* Crit Care Med 2012 4 778 786 10.1097/CCM.0b013e318236f168 22036858

[B3] Tonelli MR Curtis JR Guntupalli KK Rubenfeld GD Arroliga AC Brochard L Douglas IS Gutterman DD Hall JR Kavanagh BP Mancebo J Misak CJ Simpson SQ Slutsky AS Suffredini AF Thompson BT Ware LB Wheeler AP Levy MM An official multi-society statement: the role of clinical research results in the practice of critical care medicine Am J Respir Crit Care Med 2012 4 1117 1124 10.1164/rccm.201204-0638ST 22589312

[B4] Wunsch H Angus DC Harrison DA Linde-Zwirble WT Rowan KM Comparison of medical admissions to intensive care units in the United States and United Kingdom Am J Respir Crit Care Med 2011 4 1666 1673 10.1164/rccm.201012-1961OC 21471089

[B5] Peñuelas O Frutos-Vivar F Fernández C Anzueto A Epstein SK Apezteguía C González M Nin N Raymondos K Tomicic V Desmery P Arabi Y Pelosi P Kuiper M Jibaja M Matamis D Ferguson ND Esteban A Characteristics and outcomes of ventilated patients according to time to liberation from mechanical ventilation Am J Respir Crit Care Med 2011 4 430 437 10.1164/rccm.201011-1887OC 21616997

[B6] Frankfort-Nachmias C Leon-Guerrero A Social Statistics for a Diverse Society 2010 5 Sage Publications, Inc; Sixth Edition

[B7] R A Language Environment for Statistical Computing Vienna, Austria: R foundation for statistical computing

[B8] Baillard C Fosse J-P Sebbane M Chanques G Vincent F Courouble P Cohen Y Eledjam J-J Adnet F Jaber S Noninvasive ventilation improves preoxygenation before intubation of hypoxic patients Am J Respir Crit Care Med 2006 4 171 177 10.1164/rccm.200509-1507OC 16627862

[B9] Ventilation with lower tidal volumes as compared with traditional tidal volumes for acute lung injury and the acute respiratory distress syndrome. The Acute Respiratory Distress Syndrome Network N Engl J Med 2000 4 1301 1308 10793162 10.1056/NEJM200005043421801

[B10] Brochard L Roudot-Thoraval F Roupie E Delclaux C Chastre J Fernandez-Mondéjar E Clémenti E Mancebo J Factor P Matamis D Ranieri M Blanch L Rodi G Mentec H Dreyfuss D Ferrer M Brun-Buisson C Tobin M Lemaire F Tidal volume reduction for prevention of ventilator-induced lung injury in acute respiratory distress syndrome. The Multicenter Trail Group on Tidal Volume reduction in ARDS Am J Respir Crit Care Med 1998 4 1831 1838 10.1164/ajrccm.158.6.9801044 9847275

[B11] Dreyfuss D Saumon G Ventilator-induced lung injury: lessons from experimental studies Am J Respir Crit Care Med 1998 4 294 323 10.1164/ajrccm.157.1.9604014 9445314

[B12] Strøm T Martinussen T Toft P A protocol of no sedation for critically ill patients receiving mechanical ventilation: a randomised trial Lancet 2010 4 475 480 10.1016/S0140-6736(09)62072-9 20116842

[B13] Kress JP Pohlman AS O’Connor MF Hall JB Daily interruption of sedative infusions in critically ill patients undergoing mechanical ventilation N Engl J Med 2000 4 1471 1477 10.1056/NEJM200005183422002 10816184

[B14] Girard TD Kress JP Fuchs BD Thomason JWW Schweickert WD Pun BT Taichman DB Dunn JG Pohlman AS Kinniry PA Jackson JC Canonico AE Light RW Shintani AK Thompson JL Gordon SM Hall JB Dittus RS Bernard GR Ely EW Efficacy and safety of a paired sedation and ventilator weaning protocol for mechanically ventilated patients in intensive care (Awakening and Breathing Controlled trial): a randomised controlled trial Lancet 2008 4 126 134 10.1016/S0140-6736(08)60105-1 18191684

[B15] Prasad M Holmboe ES Lipner RS Hess BJ Christie JD Bellamy SL Rubenfeld GD Kahn JM Clinical protocols and trainee knowledge about mechanical ventilation JAMA J Am Med Assoc 2011 4 935 941 10.1001/jama.2011.1226 21900133

[B16] Cabana MD Rand CS Powe NR Wu AW Wilson MH Abboud PA Rubin HR Why don’t physicians follow clinical practice guidelines? A framework for improvement JAMA J Am Med Assoc 1999 4 1458 1465 10.1001/jama.282.15.1458 10535437

[B17] Mohan D Angus DC Thought outside the box: intensive care unit freakonomics and decision making in the intensive care unit Crit Care Med 2010 4 S637 S641 21164408 10.1097/CCM.0b013e3181f202c3

[B18] Loi numero 2002–303 du 4 mars 2002 Relative aux Droits des Malades et à la Qualité du Système de Santé Journal Officiel de la République française 5 mars 2002 4 4118 4158

[B19] Sinuff T Cook D Giacomini M Heyland D Dodek P Facilitating clinician adherence to guidelines in the intensive care unit: a multicenter, qualitative study Crit Care Med 2007 4 2083 2089 10.1097/01.ccm.0000281446.15342.74 17855822

[B20] Kahn JM Ten Have TR Iwashyna TJ The relationship between hospital volume and mortality in mechanical ventilation: an instrumental variable analysis Health Serv Res 2009 4 862 879 10.1111/j.1475-6773.2009.00959.x 19674428 PMC2699912

[B21] Lecuyer L Chevret S Guidet B Aegerter P Martel P Schlemmer B Azoulay E Case volume and mortality in hematological patients with acute respiratory failure Eur Respir J Off J Eur Soc Clin Respir Physiol 2008 4 748 754 10.1183/09031936.00142907 18448491

[B22] Lin H-C Xirasagar S Chen C-H Hwang Y-T Physician’s case volume of intensive care unit pneumonia admissions and in-hospital mortality Am J Respir Crit Care Med 2008 4 989 994 10.1164/rccm.200706-813OC 18263804

[B23] Kahn JM Goss CH Heagerty PJ Kramer AA O’Brien CR Rubenfeld GD Hospital volume and the outcomes of mechanical ventilation N Engl J Med 2006 4 41 50 10.1056/NEJMsa053993 16822995

