# Supplementary material for: Mechanical ventilation and clinical practice heterogeneity in intensive care units: a multicenter case-vignette study
Source: Ann Intensive Care. 2014 Feb 1;4:2. doi: 10.1186/2110-5820-4-2 (PMC3922080; doi:10.1186/2110-5820-4-2)

**Case-vignettes and mechanical ventilation**

*Mechanical ventilation is applied to one in two patients admitted in the intensive care unit (ICU) and is responsible for high morbidity and mortality rates. However, a large number of processes of care associated with mechanical ventilation lack proper recommendations or guidelines. Therefore, it is likely that there is a large heterogeneity in processes of care within and across different ICUs. The aim of our study is to describe the variety of practices within and across ICUs.*

*Please find the attached two clinical cases, which include 26 questions. For each question, please select the answers, which best mirror, your current practice.*

*The completion of this questionnaire should not exceed more than 15-20min.*

*On behalf of the Réseau Européen de Ventilation Artificielle (REVA), we would like to thank you in advance for your participation in this study.*

*We will update you with our results as soon as possible.*

*Sincerely*

***Yên-Lan Nguyen, Laurent Brochard, Alain Mercat et Jean-Christophe Richard on behalf of REVA.***

Do you accept to participate to this study?

🞎 Yes

🞎 If no, why?

**Case-vignette n°1:**

You are paged by the emergency department to evaluate Mr. M., 75 years old, retiree and former postman. His past medical history includes chronic obstructive pulmonary disease, morbid obesity, dyslipidemia and type 2 diabetes. He drinks alcohol occasionally. He has no known allergies. His current medications are fluticasone-salmeterol, atorvastatine and aspirin.

He presented to the emergency department because of a worsening shortness of breath over the past 5 days and insomnia last night. He has a productive cough with yellow sputum. He feels feverish. At home, he increased his dosage of fluticasone-salmeterol and added some acetaminophen.

On examination, the patient has a blood pressure of 160/80 mmHg, heart rate of 95/min, respiratory rate of 30 breaths/min and the pulse oxymetry is 92% on 5L /min O2. The patient is seated in his stretcher, breathing with pursed lips and has sweat on his forehead. On pulmonary auscultation, he had abnormal breath sounds and crackles at the right base. The patient has retrognathia. The rest of his examination was unremarkable. Blood gas performed on 5L/min O2: pH= 7.30, PaCO2= 65 mmHg, PaO2= 65mmHg, HCO3= 28 mmol/L. Chest X-ray showed a diffuse hyperlucency and an alveolar syndrome in the right base.

You decide to admit the patient to the intensive care unit to start a treatment with non-invasive ventilation, antibiotics and bronchodilators.

2 hours after ICU admission, the patient is poorly responsive with increasing hypoxemia and you think that invasive mechanical ventilation is necessary.

1. **Which physician-patient approach do you recommend? (single answer)**

a. You inform the patient that you need to intubate him without asking for his consent

b. You ask the patient for an informed consent first before taking the decision to intubate

c. You inform the family of Mr M. that his health status requires an intubation without asking for their consent

d. You ask the family for an informed consent before taking the decision to intubate

e. Other

1. **Which method of pre-oxygenation do you use? (single answer)**

a. Nasal canula

b. Simple face mask

c. Oxygen reservoir bag

d. Non-invasive ventilation

1. **Which kind of intubation do you perform? (single answer)**

a. Oral intubation

b. Nasal intubation

c. Nasal intubation with fiber optic control

1. **Which medications do you use for intubation? (one or more answers)**

a Benzodiazepines

b Ketamine

c Hypnomidate

d Morphine

e Curare

f Propofol

After, 2 attempts, you manage to intubate the patient with an introducer and you use an endotracheal tube size 8.

1. **What is your method to prevent hypotension? (one or more answers)**
   a systematic volume expansion before intubation
   b volume expansion on demand (prepared in advance)
   c systematic infusion of vasopressors
   d Infusion of vasopressors on demand (prepared in advance)
   e By other means (ventilator settings)
   f Not in this context
2. **What method do you use to check the position of the endotracheal tube? (one or more answers)**

a Visualisation with the laryngoscope

b Pulmonary auscultation

c Capnography

d Chest x-ray

e Chest x-ray if any doubt

1. **Which method do you use to assess the endotracheal cuff pressure? (one or more answers)**

a By hand

b Use of a manometer

c Checking the leakage level shown by the ventilator

1. **Do you start any sedation and/or analgesia during the first 24h? (one or more answers)**

a Continuous IV sedation

b Intermittent IV sedation

c Continuous IV analgesia

d Intermittent IV analgesia

e If needed

1. **Do you ask for restraints? (one answer)**

a Yes, systematically

b No, never

c Only if needed

1. **On the medical chart do you specify a patient position? (one answer)**

a No

b Yes, but only for a particular position (prone or lateral position)

c Yes, always, elevation of the head of the bed of 30-45°

1. **What kind of mechanical ventilation mode do you use? (one answer)**

a Volume controlled ventilation

b Assist control volume ventilation

c Intermittent assist-control ventilation

d Pressure support ventilation

e Biphasic Positive Airway Pressure (BiPAP)

f Pressure regulated volume control (e.g. PRVC, Auto-Flow)

g Other

A chest X-ray is performed after intubation.

1. **The nurse asks you whether you would like to order a chest x-ray for tomorrow. (one answer)**

a Yes, systematically, every day as long as the patient is on the ventilator

b On demand if the initial chest X-ray is abnormal

c No, we just need a chest x-ray two or three times a week

d On demand if an acute event occurs (e.g. superior vena cava central line insertion)

You would like to conduct an endotracheal suctioning.

1. **On a scale of 0 to 10, how do you evaluate the pain associated with endotracheal suctioning? (one answer)**

a 0

b 1-2

c 3-4

d 5-6

e >7

f I do not know

3 days after admission, the patient's condition improves at both clinical and biological levels. He has no other organ failure. Mr. M. is conscious and responds appropriately to simple commands. His ventilator settings are: ACV, VT=550mL, RR=16/min, PEEP=6cmH20, Fi02=0.4. You plan to start a weaning trial.

1. **What weaning test do you use? (one answer)**

a T-tube ± extubation

b Pressure support ± extubation

c Pressure support, then T-tube ± extubation

d Assist-control ventilation then T-tube ± extubation

e Assist-control ventilation, then pressure support ± extubation

f Other

1. **How long do you plan to conduct the weaning trial? (one answer)**

a < 30min

b 30min-2h

c > 2h

The weaning test is very well tolerated. You consider extubating Mr. M. who had a difficult intubation.

1. **What do you do? (one answer)**

a You extubate the patient and will give Mr. M. appropriate medicine if he presents with laryngeal edema

b You start general corticosteroids and delay extubation by 24-48h

c You conduct a cuff-leak test with the ventilator by cuff deflation and you calculate the % of leakage before considering extubating the patient

d You conduct a clinical leak test by cuff deflation and tube obstruction with a finger before considering extubating the patient

Before starting the weaning trial, Mr. M. removes his endotracheal tube.

1. **What attitude do you recommend towards reintubation? (one or more answers)**

a No indication for withholding reintubation

b You decide to not re-intubate the patient

c You discuss with the patient

d You discuss with the family

e This possibility had already been discussed since admission

**Case-vignette n°2:**

You are on night call and are paged to see Mrs. P. 30 years old, without any major medical history and hospitalized for 12 hours for a suspicion of acute respiratory distress syndrome related to H1N1 infection (viral exams pending, preliminary bacteriological exams are negative). During the patient handoff, your colleagues told you that her last blood gas at FiO2 of 0.6 showed a PaO2/FiO2 of 150. Mrs. P. is sedated with midazolam and sufentanyl.

1. **What ventilation mode do you select for Mrs. P.? (one answer)**

a Assist-control volume ventilation

b Assist-control pressure ventilation

c Pressure ventilation such as BIPAP, PRVC

d Pressure regulated volume control (e.g. PRVC, Auto-Flow))

e Other

1. **How do you set the initial tidal volume for Mrs. P.? (one answer)**

a You select 400 or 450mL because she is a woman

b You do not select a precise tidal volume but check for the peak pressure

c You do not select a precise tidal volume but check for the plateau pressure

d You ask the nurse for Mrs. P.'s weight so you can use the 6mL/Kg formula

e You measure the size of the patient and use the 6mL/Kg of predicted body weight formula

f Other

1. **Concerning the surveillance of plateau pressure, what do you do? (one answer)**

a The plateau pressure does not bother you because you are using a pressure mode

b You ask the nurse to monitor plateau pressure regularly (e.g. every 3 hours)

c You monitor peak pressure and check the plateau pressure any time you are stopping by the patient

d Other

The nurse paged you because the ventilator alarms do not stop ringing. On the monitor, you see “high respiratory rate”. The patient's rspiratory rate is 38/min. You suspect insufficient sedation.

1. **How do you evaluate the level of sedation-analgesia of Mrs. P.? (one answer)**

a Regular examination

b Sedation scale (e.g. Richmond Agitation Sedation Score, Sedation Agitation Scale, score de Ramsay)

c Analgesia scale (e.g. Behavioral Pain Score)

d Both a sedation scale and analgesia scale

e Depending on the adaptation of the patient on the ventilator

The nurse also tells you that the pulse oxymetry of the patient decreases during each tracheal suctioning.

1. **Do you recommend a systematic prevention of these episodes of desaturation once they are recognized? (one or more answers)**

a None

b Pre-oxygenation prior any tracheal suctioning

c No systematic tracheal suctioning

d Establishment of a closed suctioning system

e Recruitment maneuver after each tracheal suctioning

The respiratory status of Mrs. P. improves after 48h. That day, the patient has a Pa02/Fi02=200 with a Fi02=0.5 and PEEP=5cmH20. She does not present any sign of residual paralysis. She has no other organ failure.

1. **What do you recommend for the cessation of sedation? (one answer)**

a Progressive stop as soon as you deem appropriate, whatever the time of the day

b Progressive stop as soon as you deem appropriate, but only during daytime (and never during the night shift)

c Daily stop sedation

d Complete stop as soon as you deem appropriate, whatever the time of the day

e Complete stop as soon as you deem appropriate, but only during day time (and never during the night shift)

One week after admission, the patient is treated for a ventilator acquired pneumonia and is still sedated.

1. **Do you recommend a tracheotomy at this stage? (one answer)**

a Yes, systematically, as soon as possible

b Yes, because there is a new complication

c No, it is too early

d Depending on prognosis

One week later, the patient is still intubated because the weaning tests were inconclusive. Mrs. P. is conscious and responds appropriately to simple commands. You met the physiotherapist who asks you if he needs to see Mrs. P.

1. **What is your answer? (one answer)**

a No, thank you, her respiratory state is much better

b Yes, passive mobilization would be perfect

c Yes and could you please transfer Mrs. P. from bed to chair?

Her husband is worried about the situation because he finds his spouse depressed.

1. **What is your answer? (one or more answers)**

a To date, it is impossible to determine whether she is depressed or not

b I could start a medication

c We should not jump to conclusions concerning her mental state

d We should wait until after extubation

e Other

**Respondent :**

**Gender :**

🞎 Female

🞎 Male

**Age :**

🞎 20-30

🞎 31-40

🞎 41-50

🞎 >50

**Function:**

🞎 Professor

🞎 Attending

🞎 Fellow

🞎 Resident

| In your unit, is there any written procedure for? | Yes | No | Do not know |
| --- | --- | --- | --- |
| Weaning from mechanical ventilation |  |  |  |
| Protective ventilation |  |  |  |
| Monitoring of analgesia-sedation |  |  |  |
| Prevention of ventilator acquired pneumonia |  |  |  |
| Decisions on withholding or withdrawing therapies |  |  |  |

| In your unit is there any annual assessment of? | Yes | No | Do not know |
| --- | --- | --- | --- |
| The incidence of ventilator acquired pneumonia |  |  |  |
| The number of self-extubations |  |  |  |

Table 1: Descriptive analyses of the answers of the questionnaire:

| Question |  | n = 396 |
| --- | --- | --- |
| A | Obtained data | 393 |
|  | Missing data | 3 |
|  | Information only | 193 (49.1) |
|  | Obtaining family consent | 24 (6.1) |
|  | Obtaining patient consent | 136 (34.6) |
|  | Obtaining patient and family consent | 34 (8.7) |
|  | Other | 6 (1.5) |
| B | Obtained data | 392 |
|  | Missing data | 4 |
|  | Simple face mask | 21 (5.4) |
|  | Oxygen reservoir bag | 65 (16.6) |
|  | Non invasive ventilation | 306 (78.1) |
|  | Nasal canula | 0 (0) |
| C | Obtained data | 396 |
|  | Missing data | 0 |
|  | Oral intubation | 347 (87.6) |
|  | Nasal intubation | 14 (3.5) |
|  | Nasal intubation with fiber optic control | 35 (8.8) |
| D | Obtained data | 389 |
|  | Missing data | 7 |
|  | Hypnotic | 59 (15.2) |
|  | Hypnotic+ Morphine + Neuro-muscular blockers | 28 (7.2) |
|  | Hypnotic + Neuro-muscular blockers | 284 (73) |
|  | Hypnotic + Morphine | 17 (4.4) |
|  | Neuro-muscular blockers | 1 (0.3) |
| E | Obtained data | 394 |
|  | Missing data | 2 |
|  | Volume expansion | 165 (41.9) |
|  | Volume expansion + Vasopressors | 190 (48.2) |
|  | Vasopressors | 17 (4.3) |
|  | Other | 11 (2.8) |
|  | Not in this context | 11 (2.8) |
| F | Obtained data | 396 |
|  | Missing data | 0 |
|  | Examination | 2 (0.5) |
|  | Capnography and examination | 8 (2) |
|  | Capnography and examination and chest x-ray | 175 (44.2) |
|  | Examination and chest x-ray | 190 (48) |
|  | Capnography and chest x-ray | 9 (2.3) |
|  | Capnography | 1 (0.3) |
|  | Chest x-ray | 11 (2.8) |
| G | Obtained data | 396 |
|  | Missing data | 0 |
|  | By hand only | 235 (59.3) |
|  | By hand + manometer or leakage level | 154 (38.9) |
|  | Manometer or leakage level | 7 (1.8) |
| H | Obtained data | 395 |
|  | Missing data | 1 |
|  | Continuous | 264 (66.8) |
|  | Intermittent | 29 (7.3) |
|  | If needed | 78 (19.7) |
|  | Other | 24 (6.1) |
| I | Obtained data | 394 |
|  | Missing data | 2 |
|  | Yes, systematically | 110 (27.9) |
|  | No, never | 14 (3.6) |
|  | If needed | 270 (68.5) |
| J | Obtained data | 395 |
|  | Missing data | 1 |
|  | No | 10 (2.5) |
|  | Yes, but only for a particular position | 27 (6.8) |
|  | Yes, always | 358 (90.6) |
| K | Obtained data | 393 |
|  | Missing data | 3 |
|  | Volume ventilation other than ACV | 48 (12.2) |
|  | ACV | 254 (64.6) |
|  | Pressure ventilation | 64 (16.3) |
|  | Other | 27 (6.9) |
| L | Obtained data | 390 |
|  | Missing data | 6 |
|  | Yes, systematically | 99 (25.4) |
|  | On demand when the initial chest x-ray is pathological | 90 (23.1) |
|  | No | 30 (7.7) |
|  | On demand if an acute event occurs | 171 (43.8) |
| M | Obtained data | 395 |
|  | Missing data | 1 |
|  | 0 | 5 (1.3) |
|  | 1-2 | 30 (7.6) |
|  | 3-4 | 85 (21.5) |
|  | 5-6 | 125 (31.6) |
|  | >=7 | 100 (25.3) |
|  | I do not know | 50 (12.7) |
| N | Obtained data | 394 |
|  | Missing data | 2 |
|  | T-tube ± extubation | 40 (10.2) |
|  | Pressure support ± extubation | 121 (30.7) |
|  | Pressure support then T-tube ± extubation | 224 (56.9) |
|  | Intermittent-assist-control ventilation, then pressure support ± extubation | 3 (0.8) |
|  | Other | 6 (1.5) |
|  | Intermittent-assist-control ventilation then T-tube ± extubation | 0 (0) |
| O | Obtained data | 393 |
|  | Missing data | 3 |
|  | <30min | 66 (16.8) |
|  | 30min-2h | 281 (71.5) |
|  | >2h | 46 (11.7) |
| P | Obtained data | 387 |
|  | Missing data | 9 |
|  | Extubation and appropriate medicine | 104 (26.9) |
|  | Corticosteroids | 36 (9.3) |
|  | Cuff-leak test with the ventilator | 167 (43.2) |
|  | Cuff-leak test by hand | 80 (20.7) |
| Q | Obtained data | 395 |
|  | Missing data | 1 |
|  | No limitation | 234 (59.2) |
|  | Limitation | 8 (2) |
|  | Start discussion | 153 (38.7) |
| R | Obtained data | 394 |
|  | Missing data | 2 |
|  | Assist-control volume ventilation | 320 (81.2) |
|  | Assist-control pressure ventilation | 22 (5.6) |
|  | Pressure ventilation such as BIPAP, PRVC | 6 (1.5) |
|  | Pressure regulated volume control | 43 (10.9) |
|  | Other | 3 (0.8) |
| S | Obtained data | 394 |
|  | Missing data | 2 |
|  | You do not select a precise tidal volume but check for the plateau pressure | 39 (9.9) |
|  | Formula of 6mL/Kg | 41 (10.4) |
|  | Formula of 6mL/Kg of predicted body weight | 300 (76.1) |
|  | Other | 14 (3.6) |
| T | Obtained data | 387 |
|  | Missing data | 9 |
|  | Pressure mode | 10 (2.6) |
|  | Monitoring by nurse | 207 (53.5) |
|  | Monitoring anytime you are stopping by the room | 149 (38.5) |
|  | Other | 21 (5.4) |
| U | Obtained data | 388 |
|  | Missing data | 8 |
|  | Regular examination | 40 (10.3) |
|  | Sedation scale | 141 (36.3) |
|  | Analgesia scale | 1 (0.3) |
|  | Both a sedation scale and analgesia scale | 113 (29.1) |
|  | Depending on the adaptation of the patient on the ventilator | 93 (24) |
| V | Obtained data | 395 |
|  | Missing data | 1 |
|  | No prevention | 10 (2.5) |
|  | Prevention during suctioning only | 55 (13.9) |
|  | Global prevention | 122 (30.9) |
|  | Prevention during suctioning and global | 208 (52.7) |
| W | Obtained data | 394 |
|  | Missing data | 2 |
|  | Progressive stop | 82 (20.8) |
|  | Progressive stop during the day only | 45 (11.4) |
|  | Daily stop | 98 (24.9) |
|  | Complete stop as soon as you deem appropriate | 120 (30.5) |
|  | Complete stop as soon as you deem appropriate but during the day only | 49 (12.4) |
| X | Obtained data | 395 |
|  | Missing data | 1 |
|  | Yes, systematically | 6 (1.5) |
|  | Yes because there is this new complication | 18 (4.6) |
|  | No, it is too early | 207 (52.4) |
|  | Depending on prognosis | 164 (41.5) |
| Y | Obtained data | 387 |
|  | Missing data | 9 |
|  | No | 3 (0.8) |
|  | Yes, passive mobilization | 136 (35.1) |
|  | Yes, transfer from bed to chair | 248 (64.1) |
| Z | Obtained data | 395 |
|  | Missing data | 1 |
|  | Wait | 203 (51.4) |
|  | Medical treatment | 192 (48.6) |

#### Graphs representing the mean IQV by questions

Each bar represents the distribution of answers per question (the different colors correspond to the different answers (single answers if the question requires one single answer or combination of answers if the questions required multiple answers).

Y axis : frequencies of each answer (to simplify visualisation, we grouped the lowest frequent answers and show only 5 answers)

X axis : each letter correponds to one of the 26 questions of the case-vignettes

Above each histogram, the number corresponds to the mean IQV


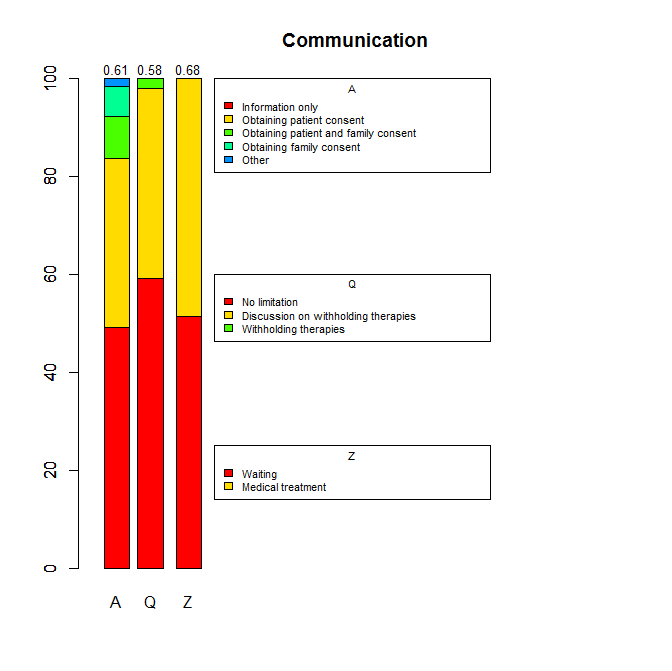


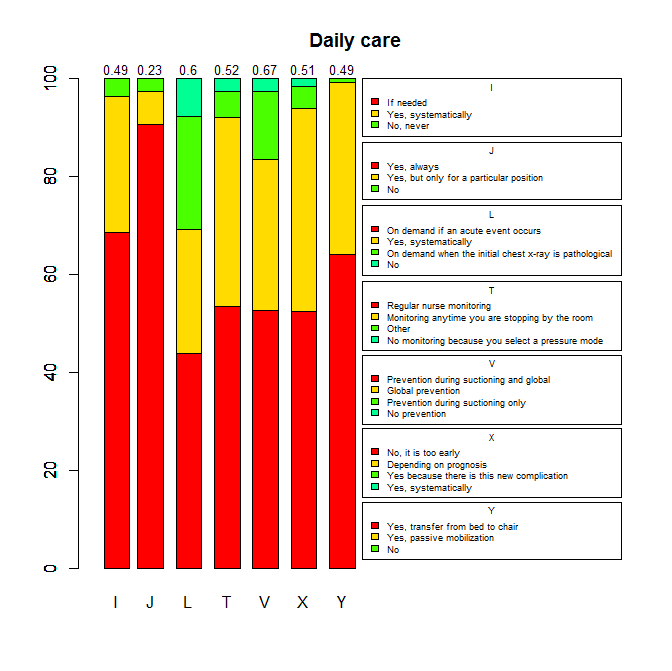


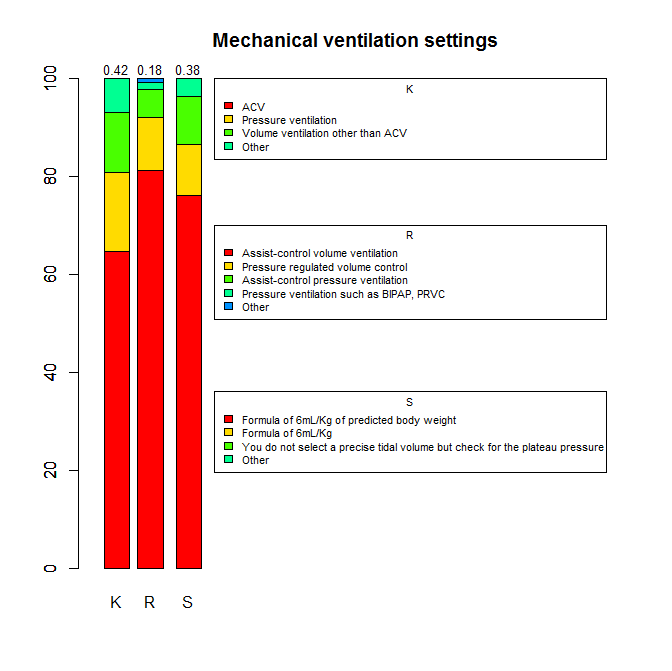


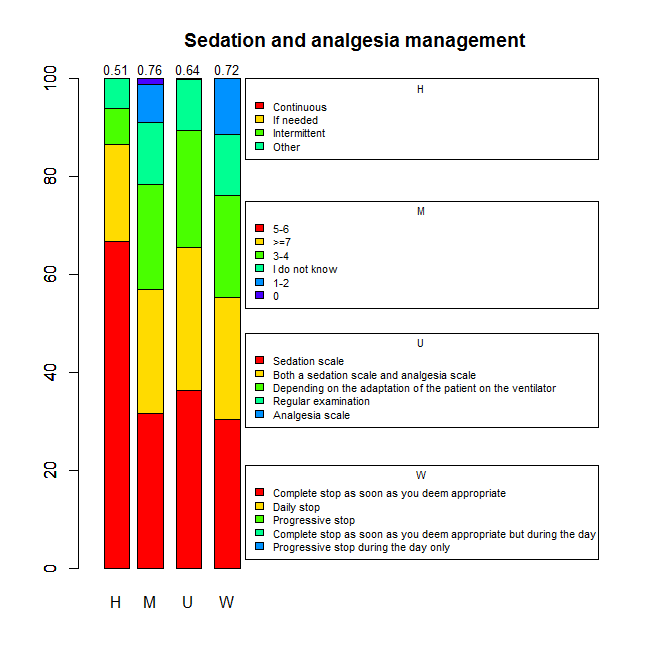


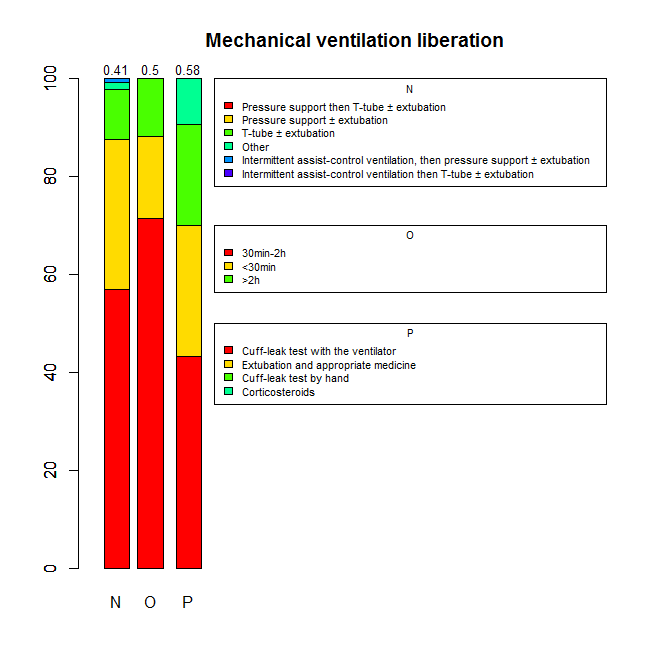


Table with the number of respondents per center

| **centres** | **respondents** |
| --- | --- |
| **1** | 8 |
| **2** | 16 |
| **3** | 6 |
| **4** | 15 |
| **5** | 6 |
| **6** | 14 |
| **7** | 8 |
| **8** | 6 |
| **9** | 13 |
| **10** | 8 |
| **11** | 4 |
| **12** | 12 |
| **13** | 18 |
| **14** | 27 |
| **15** | 9 |
| **16** | 12 |
| **17** | 5 |
| **18** | 9 |
| **19** | 12 |
| **20** | 6 |
| **21** | 15 |
| **22** | 13 |
| **23** | 11 |
| **24** | 4 |
| **25** | 8 |
| **26** | 4 |
| **27** | 6 |
| **28** | 11 |
| **29** | 6 |
| **30** | 11 |
| **31** | 17 |
| **32** | 7 |
| **33** | 7 |
| **34** | 12 |
| **35** | 7 |
| **36** | 11 |
| **37** | 8 |
| **38** | 7 |
| **39** | 9 |
| **40** | 8 |

**Graph representing the annual volume of ICU admissions per center**


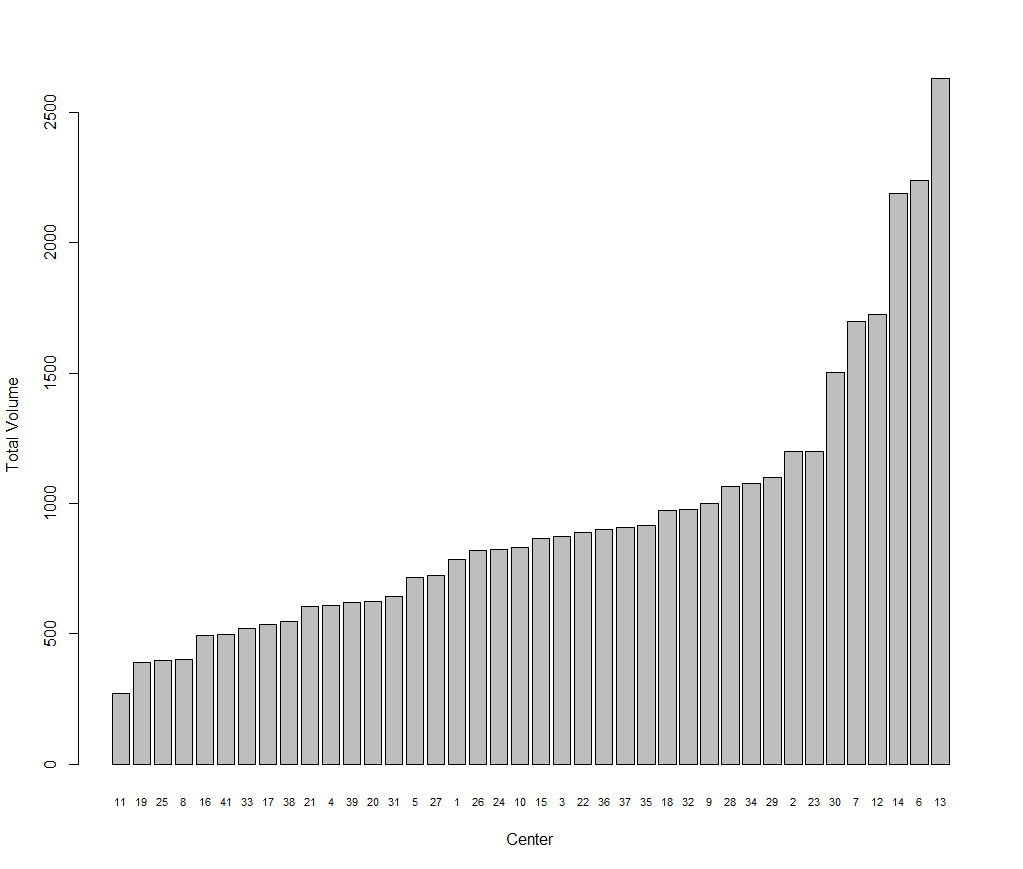


**Graph representing the annual number of MV volume admissions per center**


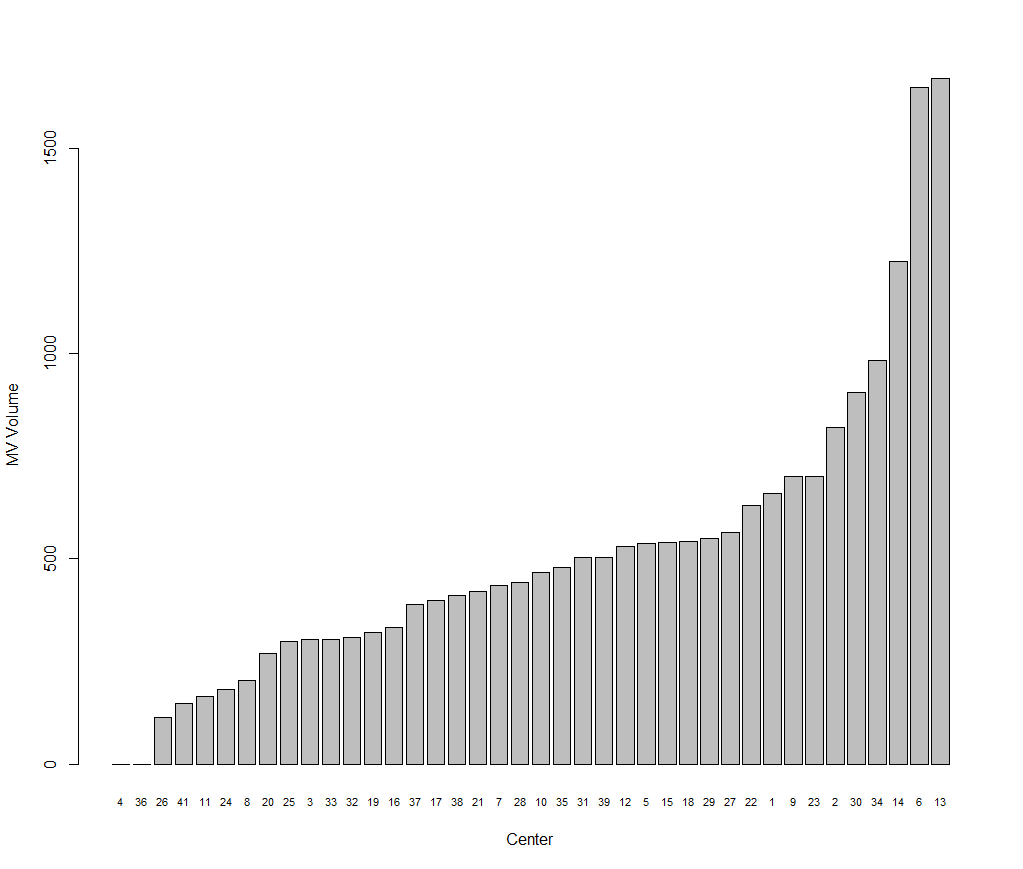


**Graph representing the annual number of admissions including those with MV**


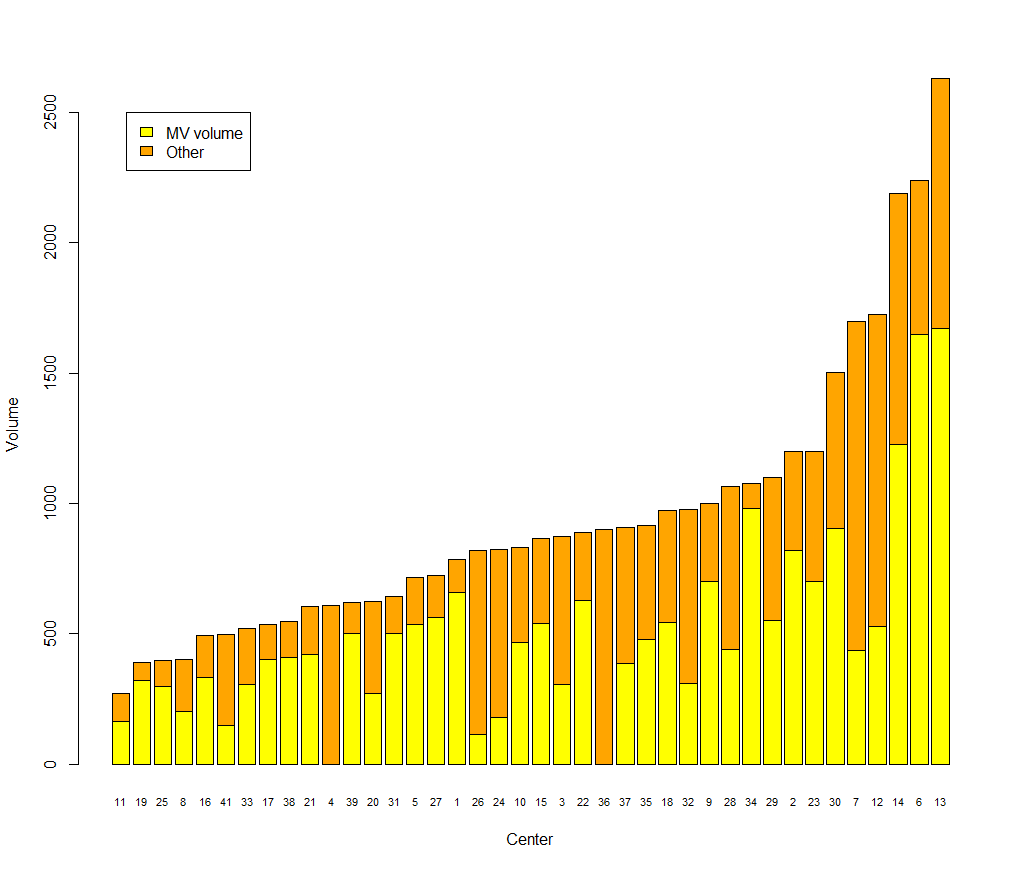


**Table representing the mean IQV per center**

| **Centre** | **IQVMean** | **IQVSD** |
| --- | --- | --- |
| **1** | 0,23 | 0,25 |
| **2** | 0,51 | 0,33 |
| **3** | 0,59 | 0,25 |
| **4** | 0,52 | 0,27 |
| **5** | 0,57 | 0,29 |
| **6** | 0,53 | 0,29 |
| **7** | 0,53 | 0,28 |
| **8** | 0,43 | 0,33 |
| **9** | 0,53 | 0,28 |
| **10** | 0,45 | 0,32 |
| **11** | 0,50 | 0,31 |
| **12** | 0,29 | 0,31 |
| **13** | 0,43 | 0,25 |
| **14** | 0,47 | 0,30 |
| **15** | 0,46 | 0,31 |
| **16** | 0,63 | 0,22 |
| **17** | 0,45 | 0,31 |
| **18** | 0,44 | 0,31 |
| **19** | 0,40 | 0,33 |
| **20** | 0,49 | 0,31 |
| **21** | 0,54 | 0,29 |
| **22** | 0,49 | 0,26 |
| **23** | 0,38 | 0,34 |
| **24** | 0,55 | 0,30 |
| **25** | 0,60 | 0,22 |
| **26** | 0,42 | 0,33 |
| **27** | 0,54 | 0,26 |
| **28** | 0,66 | 0,23 |
| **29** | 0,55 | 0,26 |
| **30** | 0,66 | 0,19 |
| **31** | 0,49 | 0,31 |
| **32** | 0,50 | 0,26 |
| **33** | 0,49 | 0,35 |
| **34** | 0,67 | 0,25 |
| **35** | 0,64 | 0,24 |
| **36** | 0,49 | 0,32 |
| **37** | 0,59 | 0,23 |
| **38** | 0,42 | 0,38 |
| **39** | 0,62 | 0,26 |
| **40** | 0,42 | 0,33 |

**Table representing the mean IQV per question**

| **Question** | **IQVMean** | **IQVSD** |
| --- | --- | --- |
| **A** | 0,61 | 0,22 |
| **B** | 0,39 | 0,31 |
| **C** | 0,26 | 0,31 |
| **D** | 0,38 | 0,29 |
| **E** | 0,55 | 0,21 |
| **F** | 0,40 | 0,24 |
| **G** | 0,64 | 0,20 |
| **H** | 0,51 | 0,27 |
| **I** | 0,49 | 0,31 |
| **J** | 0,23 | 0,31 |
| **K** | 0,42 | 0,30 |
| **L** | 0,60 | 0,28 |
| **M** | 0,76 | 0,15 |
| **N** | 0,41 | 0,25 |
| **O** | 0,50 | 0,30 |
| **P** | 0,58 | 0,28 |
| **Q** | 0,58 | 0,22 |
| **R** | 0,18 | 0,23 |
| **S** | 0,38 | 0,32 |
| **T** | 0,52 | 0,28 |
| **U** | 0,64 | 0,23 |
| **V** | 0,67 | 0,18 |
| **W** | 0,72 | 0,22 |
| **X** | 0,51 | 0,24 |
| **Y** | 0,49 | 0,29 |
| **Z** | 0,68 | 0,32 |

**Graph representing the mean IQV per question for all questions of the case vignettes (in increasing order)**


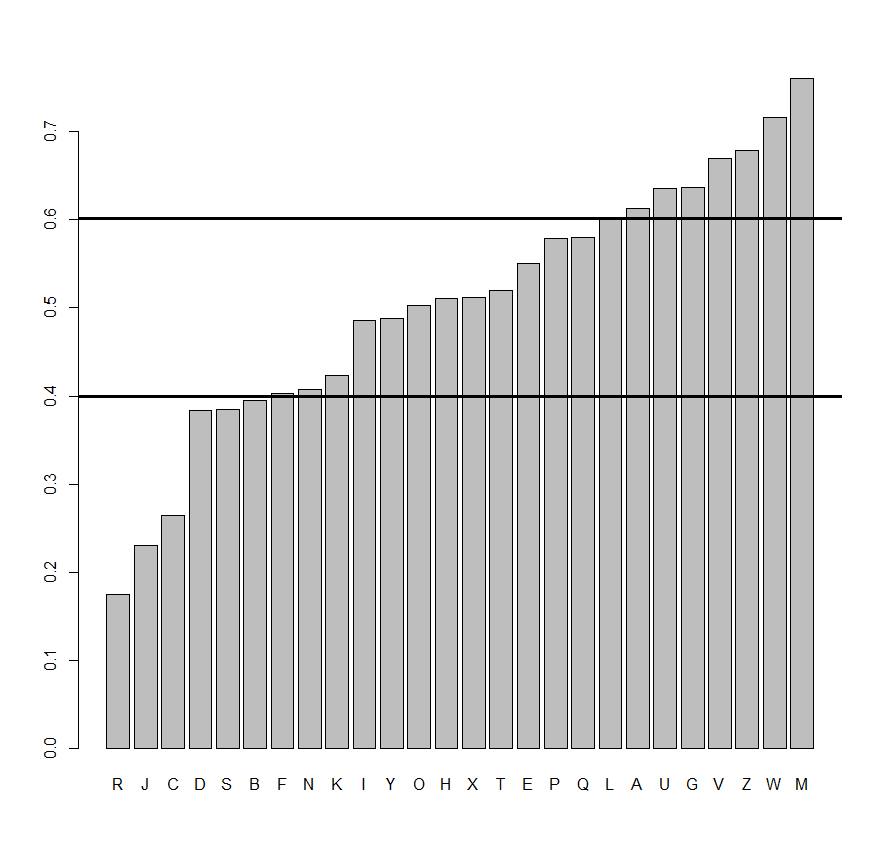


**Graph representing the association between annual volume of ICU admissions and mean IQV per center**


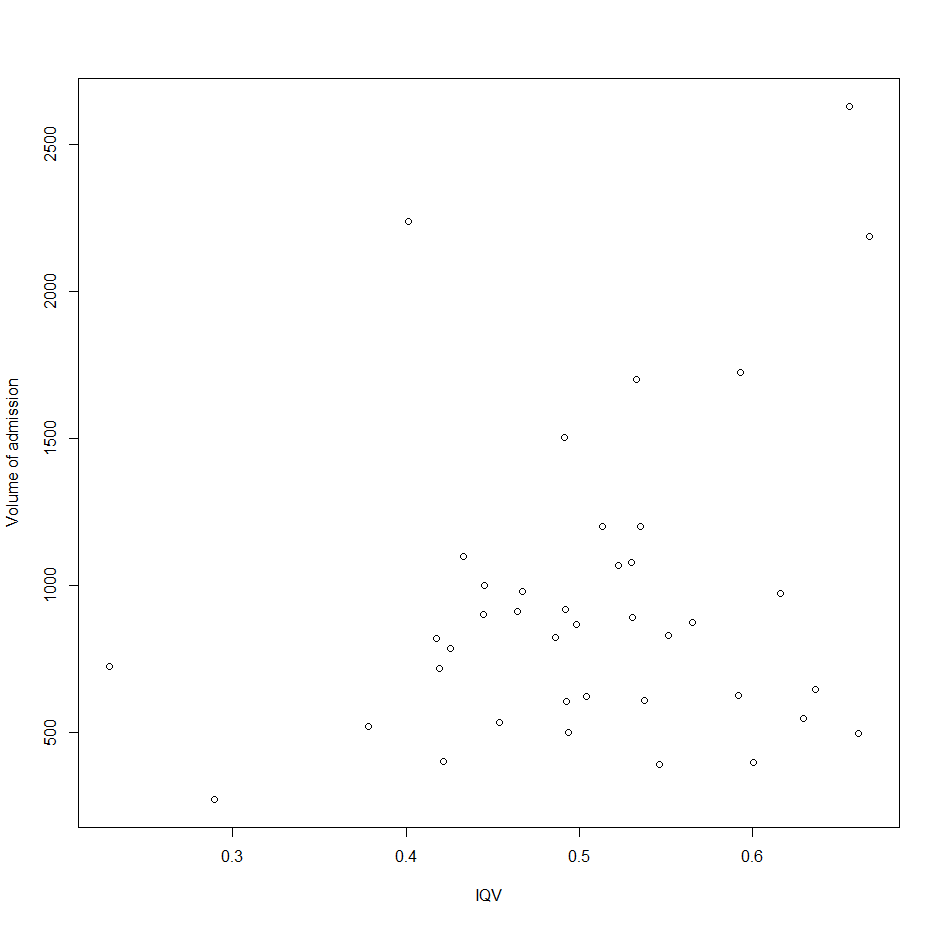


**Graph representing the association between annual volume of MV admissions and mean IQV per center**


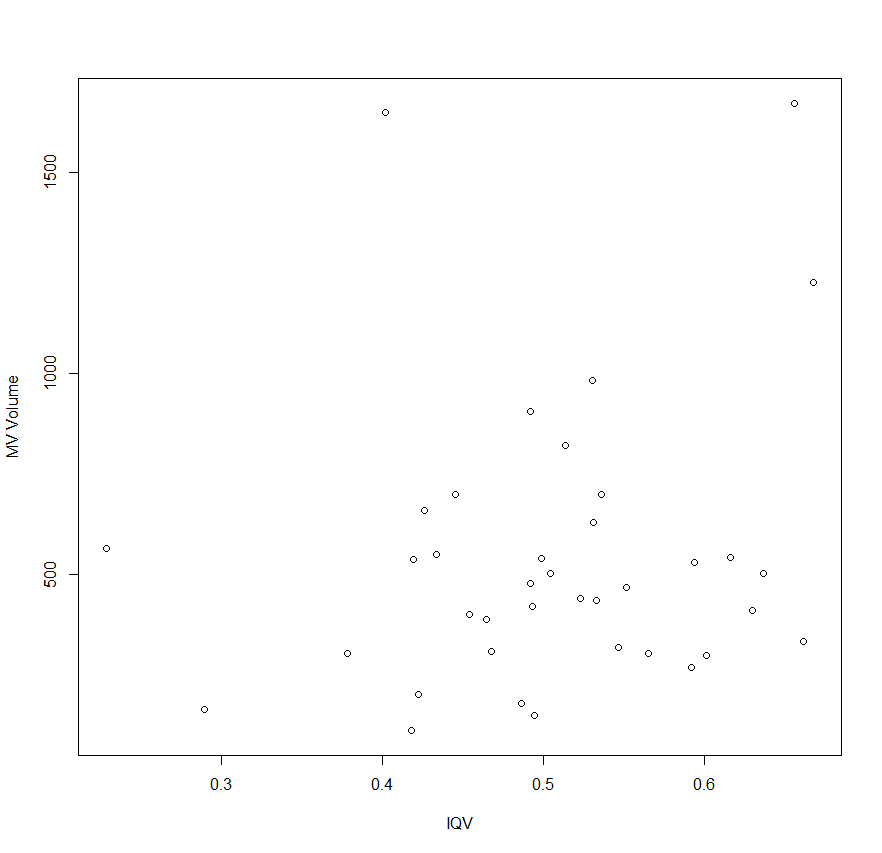

Supplement: Additional file 1 — Case-vignettes and mechanical ventilation. [file 2110-5820-4-2-S1.doc]
